# Supplementary material for: Evolving life-history traits promote biodiversity via eco-evolutionary feedback mechanisms
Source: PLoS Biol. 2025 Nov 12;23(11):e3003492. doi: 10.1371/journal.pbio.3003492 (PMC12646416; doi:10.1371/journal.pbio.3003492)
Supplement: S1 Text — (PDF) [file pbio.3003492.s001.pdf]

S1. Ecological dynamics and derivation of the fitness gradient and curvature of the fitness landscape

The model presented in the main text (eq. 1) can be rewritten into an equivalent model in terms of total density $N_j = A_j + J_j$, and the fraction of adults $C_j = A_j/N_j$. Changes in the variable C_j therefore reflect changes in the population composition. The dynamics of the population densities, fraction of adults, and food resources thus follow

$$\frac{dN_j}{dt} = \left[\beta_j(\eta_j, \ell_j, \mathrm{F})C_j - \left(\delta_J(\ell_j)(1 - C_j) + \delta_A C_j\right)\right]N_j$$

$$\frac{dC_j}{dt} = \Phi_j(\eta_j, \ell_j, \mathrm{F})(1 - C_j) - \beta_j(\eta_j, \ell_j, \mathrm{F}){C_j}^2 - \left(\delta_A - \delta_J(\ell_j)\right)(1 - C_j)C_j,$$

$$\frac{dF_i}{dt} = \rho(F_{i\,max} - F_i) - \sum_{j=1}^{m} a_i(\eta_j)F_i\left(\gamma(1 - C_j) + C_j\right)N_j.$$

(S1.1)

Using the adaptive dynamics framework[1,2], it is possible to derive expressions for the fitness gradient and the curvature of the fitness landscape. The relative fitness of a rare mutant population with phenotype $\mathrm{x}_j' = (\eta_j', \ell_j')$ depends on the environment set by ecomorph populations with trait values $\mathbf{x} = (\mathrm{x}_1, \dots, \mathrm{x}_m)$. Therefore, we evaluate the relative fitness of a rare mutant x_j' at ecological equilibrium when resident ecomorphs have traits $\mathbf{x}$:

$$W(\mathrm{x}_j', \mathbf{x}) = \beta_j\left(\eta_j', \ell_j', \mathrm{F}(\mathbf{x})\right) C_j(\ell_j') - \left(\delta_J(\ell_j')(1 - C_j(\ell_j')) + \delta_A C_j(\ell_j')\right)$$

(S1.2)

This is because the population composition C_j does depend on the life history trait but not on the feeding niche trait (see eq. SI3.16).

Under the assumption that evolution occurs via small, infrequent mutational steps, the evolutionary trajectory can be approximated by the canonical equation of adaptive dynamics[1,2]:

$$\frac{d\mathrm{x}_j}{dt} = \mathrm{M}(\mathrm{x}_j)\,\mathrm{A}(\mathrm{x}_j)\,\nabla W_j(\mathrm{x}_j, \mathbf{x})$$

(S1.3)

In this expression, $\mathrm{M}(\mathrm{x}_j)$ is a function describing variation in the rate of the occurrence of mutations (e.g. due to variation in population size), $\mathrm{A}(\mathrm{x}_j)$ is the mutational variance-covariance matrix summarizing the distribution of mutations around the phenotype x_j, and $\nabla W_j(\mathrm{x}_j, \mathbf{x})$ is the selection gradient, which is a two-dimensional vector whose components are:

$$\left.\frac{\partial W_j(\mathrm{x}_j',\mathbf{x})}{\partial \eta_j'}\right|_{\mathrm{x}_j'=\mathrm{x}_j} = \frac{\varepsilon C_j(\ell_j')}{\ell_j'}\sum_{i=1}^{n}\frac{\alpha\,(\theta_i-\eta_j')}{\tau^2}\exp\left[\frac{-(\theta_i-\eta_j')^2}{2\tau^2}\right]F_i(\mathbf{x})$$

(S1.4)

$$\left.\frac{\partial W_j(\mathrm{x}_j',\mathbf{x})}{\partial \ell_j'}\right|_{\mathrm{x}_j'=\mathrm{x}_j}$$
$$= \frac{\varepsilon}{\ell_j'}\left(\frac{\partial C_j}{\partial \ell_j'} - \frac{C_j(\ell_j')}{\ell_j'}\right)\sum_{i=1}^{n} a_i(\eta_j')F_i(\mathbf{x}) - \frac{\nu}{\ell_j'}\left(\frac{\partial C_j}{\partial \ell_j'} - \frac{C_j(\ell_j')}{\ell_j'}\right)$$
$$+ \delta_{\max}e^{-\ell_j'}\left(1 - C_j(\ell_j') + \frac{\partial C_j}{\partial \ell_j'}\right) - \delta_A\frac{\partial C_j}{\partial \ell_j'}$$

(S1.5)

Diversification occurs through a process of evolutionary branching when directional selection in the feeding niche trait, halts, if this trait value is a minimum of the fitness landscape [3,4]. The curvature of the fitness function therefore determines whether an ecomorph splits into two different ecomorphs. Hence, the second derivative of the first component of the fitness function with respect to η_j' evaluated at the ecological equilibrium when ecomorphs have traits $\mathbf{x}$ allows us to determine whether a diversification event occurs

$$\left.\frac{\partial^2 W_j(\mathrm{x}_j',\mathbf{x})}{\partial {\eta_j'}^2}\right|_{\mathrm{x}_j'=\mathrm{x}_j} = \frac{\varepsilon C_j(\ell_j')}{\ell_j'}\sum_{i=1}^{n}\frac{\alpha\ \left((\theta_i-\eta_j')^2-\tau^2\right)}{\tau^4}\exp\left[\frac{-(\theta_i-\eta_j')^2}{2\tau^2}\right]F_i(\mathbf{x}).$$

(S1.6)

References

1. Dieckmann, U. & Law, R. *J. Math. Biol.* **34**, 579–612 (1996).
2. Leimar, O. *Evol. Ecol. Res.* **11**, 191–208 (2009).
3. Dieckmann, U. (Cambridge University Press., 2004). doi:10.1017/CBO9781139342179
4. Geritz, S. A. H. & Kisdi, E. *Proc. R. Soc. B Biol. Sci.* **267**, 1671–1678 (2000).
