# Supplementary material for: Evolving life-history traits promote biodiversity via eco-evolutionary feedback mechanisms
Source: PLoS Biol. 2025 Nov 12;23(11):e3003492. doi: 10.1371/journal.pbio.3003492 (PMC12646416; doi:10.1371/journal.pbio.3003492)
Supplement: S2 Text — (PDF) [file pbio.3003492.s002.pdf]

S2. Analytical derivation of the ecological equilibrium

Here, we derive expressions for the ecological equilibrium when a population with total density N, fraction of adults C, feeding niche trait η, and offspring size ℓ colonizes an environment with two different food resources, F_1 and F_2. The analytical investigation is restricted to the simplest case, in which $F_{1max} = F_{2max} = F_{max}$.

From eq. S1.1 in S1, the population density, fraction of juveniles and resources satisfy the following equations at ecological equilibrium

$$0 = C\left(\varepsilon(a_1(\eta)F_1 + a_2(\eta)F_2) - v\right)/\ell - \delta_J(\ell)(1-C) - \delta_A C) \quad \text{(S2.1)}$$

$$0 = \Phi(\eta, \ell, F_1, F_2)(1-C) - \left(\varepsilon(a_1(\eta)F_1 + a_2(\eta)F_2) - v\right)C^2/\ell - (\delta_A - \delta_J(\ell))(1-C)C \quad \text{(S2.2)}$$

$$0 = \rho(F_{max} - F_1) - a_1(\eta)F_1\left(\gamma(1-C) + C\right)N \quad \text{(S2.3)}$$

$$0 = \rho(F_{max} - F_2) - a_2(\eta)F_2\left(\gamma(1-C) + C\right)N \quad \text{(S2.4)}$$

Equations S2.1, S2.3, S2.4 can be reformulated to obtain

$$0 = E(a_1(\eta)F_1 + a_2(\eta)F_2) - G \quad \text{(S2.5)}$$

$$0 = \rho(F_{max} - F_1) - a_1(\eta)F_1 IN \quad \text{(S2.6)}$$

$$0 = \rho(F_{max} - F_2) - a_2(\eta)F_2 IN \quad \text{(S2.7)}$$

where $E = \varepsilon C/\ell$, $G = vC/\ell + \delta_A C + \delta_J(1-C)$, and $I = \gamma(1-C) + C$.

Equations (S2.6) and (S2.7) give

$$F_1 = \frac{\rho F_{max}}{\rho + a_1(\eta)IN} \text{ and } F_2 = \frac{\rho F_{max}}{\rho + a_2(\eta)IN} \quad \text{(S2.8)}$$

By substituting F_1 and F_2 in equation (S2.5), an equation for N is obtained:

$$0 = E\left(a_1(\eta)\frac{\rho F_{max}}{\rho + a_1(\eta)IN} + a_2(\eta)\frac{\rho F_{max}}{\rho + a_2(\eta)IN}\right) - G$$

which is equivalent to the quadratic equation

$$0 = \alpha_N N^2 + \beta_N N + \gamma_N \quad \text{(S2.9)}$$

where

$$\alpha_N = GI^2 a_1(\eta)a_2(\eta) \quad \text{(S2.10)}$$

$$\beta_N = \rho GI(a_1(\eta) + a_2(\eta)) - 2EI\rho F_{max}a_1(\eta)a_2(\eta) \quad \text{(S2.11)}$$

$$\gamma_N = \rho^2 G - E\rho^2 F_{max}(a_1(\eta) + a_2(\eta)) \quad \text{(S2.12)}$$

The two solutions of equation (S2.9) are

$$N^*(\eta) = \frac{-\beta_N \pm \sqrt{\Delta_N}}{2\alpha_N}$$

where $\Delta_N = \beta_N^2 - 4\alpha_N\gamma_N = G^2\rho^2 I^2(a_1(\eta) - a_2(\eta))^2 + 4E^2I^2\rho^2F_{max}^2 a_1(\eta)^2 a_2(\eta)^2$. Only when $\sqrt{\Delta_N}$ is added to $-\beta_N$ the population density is positive, therefore, the only biologically relevant solution is

$$N^*(\eta) = \frac{-\beta_N + \sqrt{\Delta_N}}{2\alpha_N} \tag{S2.13}$$

By substituting N^* in equations (S2.8), it can be obtained

$$F_1^*(\eta) = \frac{2a_2(\eta)\rho F_{max}GI}{\sqrt{\rho^2(4B^2 + G^2I^2(a_2(\eta) - a_1(\eta))^2)} + \rho(2B + GI(a_2(\eta) - a_1(\eta)))} \tag{S2.14}$$

$$F_2^*(\eta) = \frac{2a_1(\eta)\rho F_{max}GI}{\sqrt{\rho^2(4B^2 + G^2I^2(a_2(\eta) - a_1(\eta))^2)} + \rho(2B - GI(a_2(\eta) - a_1(\eta)))}, \tag{S2.15}$$

where $B = EIF_{max}a_1(\eta)a_2(\eta)$.

By replacing the maturation function $\Phi(\eta, \ell, F_1, F_2)$ into equation (S2.3) one obtains:

$$0 = \left(\frac{\varepsilon\gamma(a_1(\eta)F_1 + a_2(\eta)F_2) - v - \delta_J(\ell)}{1 - \left(\frac{\ell}{w}\right)^{1 - \frac{\delta_J(\ell)}{\varepsilon\gamma(a_1(\eta)F_1 + a_2(\eta)F_2) - v}}} \right)(1-C) - (\varepsilon(a_1(\eta)F_1 + a_2(\eta)F_2) - v)\frac{C^2}{\ell} - (\delta_J - \delta_A)(1-C)C$$

From equation (S2.5), we get that in the ecological equilibrium $a_1(\eta)F_1 + a_2(\eta)F_2 = \frac{\ell G}{\varepsilon C}$. Therefore, this equation can be reformulated as

$$0 = \left(\frac{\gamma\frac{G\ell}{C} - v - \delta_J(\ell)}{1 - \left(\frac{\ell}{w}\right)^{1 - \frac{\delta_J(\ell)}{\gamma\frac{\ell G}{C} - v}}} \right)(1 - C) - \left(\frac{G\ell}{C} - v\right)\frac{C^2}{\ell} - (\delta_A - \delta_J(\ell))(1 - C)C \tag{S2.16}$$

Unfortunately, this expression cannot be solved analytically due to the complexity of the maturation function. Therefore, we cannot obtain an analytical expression for the fraction of adults C^* in the ecological equilibrium. However, from equation S2.16 it is possible to infer that the fraction of adults C^* in the ecological equilibrium does not depend on the trait η.
