# Supplementary material for: Evolving life-history traits promote biodiversity via eco-evolutionary feedback mechanisms
Source: PLoS Biol. 2025 Nov 12;23(11):e3003492. doi: 10.1371/journal.pbio.3003492 (PMC12646416; doi:10.1371/journal.pbio.3003492)
Supplement: S4 Text — (PDF) [file pbio.3003492.s004.pdf]

S4. Analytical proof that the minimum productivity required for diversification occurs when the life history trait is at its optimum

Here, we show that the threshold of productivity required for diversification takes the lowest value when the selection gradient of the life history trait (offspring size) vanishes, or in other words, when this trait is at its optimum. As in the previous sections, we consider a population with total density N, fraction of adults C, and phenotype $x = (\eta, \ell)$, where η is the feeding niche trait, and ℓ is the offspring size. The population colonizes an environment with two different food resources, F_1 and F_2. Using the adaptive dynamics framework, which assumes that evolution occurs much more slowly than the ecological dynamics, and thus that the system reaches its ecological attractor in between two mutations, we derive an expression for the offspring size when the selection gradient vanishes. Then, we calculate the minimum productivity required for diversification as a function of the offspring size.

1. Optimal offspring size From eq. S1.5, the selection gradient of a rare mutant with offspring size ℓ' is

$$\frac{\partial W}{\partial \ell'}(x',x) = \frac{\varepsilon}{\ell'}\left(\frac{\partial C}{\partial \ell'} - \frac{C}{\ell'}\right)(a_1(\eta)F_1 + a_2(\eta)F_2) - \frac{v}{\ell'}\left(\frac{\partial C}{\partial \ell'} - \frac{C}{\ell'}\right) + \delta_{max}e^{-\ell'}\left(1 - C + \frac{\partial C}{\partial \ell'}\right) - \delta_A\frac{\partial C}{\partial \ell'} \tag{S4.1}$$

Because in the ecological equilibrium $a_1(\eta)F_1 + a_2(\eta)F_2 = \frac{G}{E}$ (see eq. S2.5), we have

$$\begin{aligned}\frac{\partial W}{\partial \ell'}(x',x) &= \frac{G}{C}\frac{\partial C}{\partial \ell'} - \frac{G}{\ell'} - \frac{v}{\ell}\frac{\partial C}{\partial \ell'} + \frac{vC}{\ell'^2} + \delta_{max}e^{-\ell'}(1-C) + \delta_{max}e^{-\ell'}\frac{\partial C}{\partial \ell'} - \delta_A\frac{\partial C}{\partial \ell'}\\ &= \left(\frac{G}{C} - \frac{v}{\ell'} + \delta_{max}e^{-\ell'} - \delta_A\right)\frac{\partial C}{\partial \ell'} - \frac{G}{\ell'} + \frac{vC}{\ell'^2} + \delta_{max}e^{-\ell'}(1-C).\end{aligned} \tag{S4.2}$$

Replacing $G = \frac{vC}{\ell} + \delta_A C + \delta_{max}e^{-\ell}(1-C)$, we get

$$\begin{aligned}\frac{\partial W}{\partial \ell'}(x',x) = &\left(\frac{1}{C}\left(\frac{vC}{\ell'} + \delta_A C + \delta_{max}e^{-\ell'}(1-C)\right) - \frac{v}{\ell'} + \delta_{max}e^{-\ell'} - \delta_A\right)\frac{\partial C}{\partial \ell'}\\ &-\frac{1}{\ell'}\left(\frac{vC}{\ell'} + \delta_A C + \delta_{max}e^{-\ell'}(1-C)\right) + \frac{vC}{\ell'^2} + \delta_{max}e^{-\ell'}(1-C)\\ =&\frac{\delta_{max}e^{-\ell'}}{C}\frac{\partial C}{\partial \ell'} + \frac{1}{\ell'}\left(-\delta_A C + \delta_{max}e^{-\ell'}(1-C)(\ell'-1)\right)\end{aligned} \tag{S4.3}$$

The trait value at which the selection gradient equals zero is the optimal value for the offspring size. Therefore, this trait value can be found by solving this equation for ℓ:

$$0 = \frac{\delta_{max}e^{-\ell}}{C}\frac{\partial C}{\partial \ell} + \frac{1}{\ell}\left(-\delta_A C + \delta_{max}e^{-\ell}(1-C)(\ell-1)\right). \tag{S4.4}$$

However, it is not possible to obtain an analytical solution for this equation because we do not have an analytical expression for C and $\frac{\partial C}{\partial \ell}$ (see S2.16).

2. Minimum productivity for diversification From eq. S3.21, we know that the threshold of productivity required for diversification P_{min} is

$$P_{min} = \frac{\rho G D^2}{4\tau^2 E\alpha} e^{\frac{D^2}{8\tau^2}} \tag{S4.5}$$

Replacing the terms G and E (see eq. S2.5), P_{min} can be expressed as

$$\begin{aligned} P_{min} &= \frac{\ell}{\varepsilon C}\left(\frac{vC}{\ell} + \delta_A C + \delta_{max} e^{-\ell}(1-C)\right)\frac{\rho D^2}{4\tau^2\alpha} e^{\frac{D^2}{8\tau^2}} \\ &= \left(\frac{v}{\varepsilon} + \frac{\ell\delta_A}{\varepsilon} + \frac{\ell\delta_{max} e^{-\ell}}{\varepsilon C} - \frac{\ell\delta_{max} e^{-\ell}}{\varepsilon}\right)\frac{\rho D^2}{4\tau^2\alpha} e^{\frac{D^2}{8\tau^2}} \end{aligned} \tag{S4.6}$$

To understand how P_{min} depends on ℓ, we take the derivative of this expression with respect to ℓ

$$\frac{\partial P_{min}}{\partial \ell} = \left(\frac{\delta_A}{\varepsilon} - \frac{\delta_{max} e^{-\ell}}{\varepsilon} + \frac{\ell\delta_{max} e^{-\ell}}{\varepsilon} + \frac{\delta_{max} e^{-\ell}}{\varepsilon C} - \frac{\ell\delta_{max} e^{-\ell}}{\varepsilon C} - \frac{\ell\delta_{max} e^{-\ell}}{\varepsilon C^2}\frac{\partial C}{\partial \ell}\right)\frac{\rho D^2}{4\tau^2\alpha} e^{\frac{D^2}{8\tau^2}} \tag{S4.7}$$

Then, we set this function to zero to find the minimum

$$\begin{aligned} 0 &= \delta_A C - \delta_{max} e^{-\ell} C + \ell\delta_{max} e^{-\ell} C + \delta_{max} e^{-\ell} - \ell\delta_{max} e^{-\ell} - \frac{\ell\delta_{max} e^{-\ell}}{C}\frac{\partial C}{\partial \ell} \\ &= \delta_A C - \delta_{max} e^{-\ell}(1-C)(\ell-1) - \frac{\ell\delta_{max} e^{-\ell}}{C}\frac{\partial C}{\partial \ell} \\ &= \frac{1}{\ell}\left(-\delta_A C + \delta_{max} e^{-\ell}(1-C)(\ell-1)\right) + \frac{\delta_{max} e^{-\ell}}{C}\frac{\partial C}{\partial \ell} \end{aligned} \tag{S4.8}$$

Therefore, the threshold of minimum productivity required for diversification takes its lowest value when the offspring size takes the value of ℓ that satisfies this equation. Because this expression is equivalent to the expression for the optimal offspring size in equation (S4.4), we can affirm that the threshold of productivity required for diversification takes its lowest value when the offspring size is at the optimal value.
