# Supplementary material for: Evolving life-history traits promote biodiversity via eco-evolutionary feedback mechanisms
Source: PLoS Biol. 2025 Nov 12;23(11):e3003492. doi: 10.1371/journal.pbio.3003492 (PMC12646416; doi:10.1371/journal.pbio.3003492)
Supplement: S5 Text — Table A. Variables and parameters of Model 1. (PDF) [file pbio.3003492.s005.pdf]

S5. Parameterization of Model 1

The model presented in the main text is based on previous models of ecological diversification (e.g. ref [1–3]). In particular, we extend the stage-structured model studied by Chaparro-Pedraza[3] and adopt the values for the environmental parameters and demographic parameters independent of stage structure (i.e. α, τ, ε, ν) used in that study. Some of these values are key for diversification and thus selected accordingly, for example, τ and the distance between the two optima to feed on the food resources D must satisfy the condition $D > 2\tau$ to ensure that selection is disruptive at the trait value in between the two optima (see S3, eq. S3.6). Additionally, for the stage-dependent demographic parameters we chose values that broadly represent organisms in nature. The general rule across animal taxa is that larger organisms have higher survival[4–15] and higher foraging capacity[16–18] than smaller conspecifics. Hence, stage-specific parameters are assigned values that result in a higher survival and foraging capacity of adults than juveniles (i.e. $\delta_A < \delta_J$ and $\gamma < 1$). $\gamma = 0.5$ is adopted as the default value for the ingestion rate of juveniles, similar to values previously used in similar stage-structured models (e.g. ref [19]). Parameter values are summarized in table A.

Table A. Variables and parameters of Model 1

Variable or parameter	Symbol	Value	Units
Variables			
Feeding niche trait	η	Evolving trait	-
Offspring size	ℓ	Evolving trait	g
Individual density	N_j	-	L^{-1}
Adult fraction	C_j	-	-
Food resource density	F_i	-	g L^{-1}
Environmental parameters			
Renewal rate of resources	ρ	0.01	(unit of time)$^{-1}$
Total productivity of the habitat	P	0.4 (by default) varied in Fig. 4	g L^{-1} (unit of time)$^{-1}$
Number of resources	n	10	-
Distance between optima to feed on the resources	D	1	-
Demographic parameters			
Maximum attack rate	α	0.2	L (unit of time)$^{-1}$
Width of the Gaussian curve describing the degree of specialization to successfully attack a food resource	τ	1/3	-
Assimilation efficiency	ε	0.6	-
Metabolic cost	ν	0.01	(weight)*(unit of time)$^{-1}$
Juvenile foraging capacity factor	γ	0.5	-
Adult mortality rate	δ_A	0.01	(unit of time)$^{-1}$
Maximum juvenile mortality rate	$\delta_{\max}$	0.1 (by default) varied in Fig. 4	(unit of time)$^{-1}$
Size at maturation	w	10	g
Evolutionary parameters			
Mutation rate of the feeding niche trait	μ_η	10^{-3}	-
Mutation rate of the offspring size	μ_ℓ	10^{-3} (by default) varied in Fig. 3	-
Variance of the trait offspring distribution (feeding niche trait)	σ_η^2	0.01	-
Variance of the trait offspring distribution (offspring size)	σ_ℓ^2	0.01	-

References

1. Chaparro-Pedraza, C., Roth, G. & Melian, C. Ecological diversification in sexual and asexual lineages. *bioRxiv* 2024.03.06.583698 (2024) doi:10.1101/2024.03.06.583698.
2. Chaparro-Pedraza, P. C., Roth, G. & Seehausen, O. The enrichment paradox in adaptive radiations: Emergence of predators hinders diversification in resource rich environments. *Ecol. Lett.* **25**, 802–813 (2022).
3. Chaparro-Pedraza, P. C. Differential stage-specific mortality as a mechanism for diversification. *Am. Nat.* **(in press)**, (2024).
4. Sogard, S. M. Size selective mortality in the juvenile stages of teleost fishes: a review. *Bull. Mar. Sci.* **60**, 1129–1157 (1997).
5. Krause, J., Loader, S. P., McDermott, J. & Ruxton, G. D. Refuge use by fish as a function of body length-related metabolic expenditure and predation risks. *Proc. R. Soc. B Biol. Sci.* **265**, 2373–2379 (1998).
6. Boulton, A. M. & Polis, G. A. Phenology and Life History of the Desert Spider, Diguetia mojavea (Araneae , Diguetidae). *J. Arachnol.* **27**, 513–521 (1999).
7. Keller, G. & Ribi, G. Fish predation and offspring survival in the prosobranch snail Viviparus ater. *Oecologia* **93**, 493–500 (1993).
8. Hampton, J. Natural mortality rates in tropical tunas: size really does matter. *Can. J. Fish. Aquat. Sci.* **57**, 1002–1010 (2000).
9. Arendt, J. D. Influence of sprint speed and body size on predator avoidance in New Mexican spadefoot toads (Spea multiplicata). *Oecologia* **159**, 455–461 (2009).
10. Semlitsch, R. D. Effects of body size, sibship, and tail injury on the susceptibility of tadpoles to dragonfly predation. *Can. J. Zool.* **68**, 1027–1030 (1990).
11. Rudolf, V. H. W. Impact of Cannibalism on Predator-Prey Dynamics : Size-Structured Interactions and Apparent Mutualism. *Ecology* **89**, 1650–1660 (2008).
12. Keren-Rotem, T., Bouskila, A. & Geffen, E. Ontogenetic habitat shift and risk of cannibalism in the common chameleon (Chamaeleo chamaeleon). *Behav. Ecol. Sociobiol.* **59**, 723–731 (2006).
13. Ferguson, G. W. & Fox, S. F. Annual Variation of Survival Advantage of Large Juvenile Side-Blotched Lizards, Uta stansburiana: Its Causes and Evolutionary Significance. *Evolution (N. Y).* **38**, 342–349 (1984).
14. Tucker, J. K., Filoramo, N. I. & Janzen, F. J. Size-biased mortality due to predation in a nesting freshwater turtle, Trachemys scripta. *Am. Midl. Nat.* **141**, 198–203 (1999).
15. Rudolf, V. H. W. & Armstrong, J. Emergent impacts of cannibalism and size refuges in prey on intraguild predation systems. *Oecologia* **157**, 675–686 (2008).
16. Gribbin, S. D. & Thompson, D. J. Asymmetric intraspecific competition among larvae of the damselfly Ischnura elegans (Zygoptera: Coenagrionidae). *Ecol. Entomol.* **15**, 37–42 (1990).
17. McPeek, M. A. & Crowley, P. H. The effects of density and relative size on the aggressive behaviour, movement and feeding of damselfly larvae (Odonata: Coenagrionidae). *Anim. Behav.* **35**, 1051–1061 (1987).
18. Nakayama, S. & Fuiman, L. A. Body size and vigilance mediate asymmetric interference competition for food in fish larvae. *Behav. Ecol.* **21**, 708–713 (2010).
19. de Roos, M. Dynamic population stage structure due to juvenile – adult asymmetry stabilizes complex ecological communities. *Proc. Natl. Acad. Sci.* **118**, 1–8 (2021).
