# Supplementary material for: Evolving life-history traits promote biodiversity via eco-evolutionary feedback mechanisms
Source: PLoS Biol. 2025 Nov 12;23(11):e3003492. doi: 10.1371/journal.pbio.3003492 (PMC12646416; doi:10.1371/journal.pbio.3003492)
Supplement: S6 Text — Table B. Life history parameters of Model 2 (in S6 Text). Table C. Life history parameters of the non-dimensionalized Model 3. Figure A: Energy allocation and body size at maturation. (PDF) [file pbio.3003492.s006.pdf]

S6. Description and parameterization of Model 2 and Model 3

In this supplementary note, we describe the size-structured population models used to test the generality of our findings. The core part of these models is the description of the individual behavior of the consumer ecomorphs, that is, individual feeding, growth, development, reproduction and mortality, as a function of the current state of both the environment (e.g. resource availability) and the individual itself (i.e. its body size).

Model 2: Diversification and evolution of maturation size

The model follows the food web bioenergetic approach for size-structured populations introduced by Hartvig[1]. Here we only provide a concise synopsis of the model. We consider an ecomorph population j to be composed of individuals characterized by their feeding niche trait η_j, body size s and body size at maturation s_m. The rate at which an individual encounters food depends on η_j, s and the densities of the resources $\mathrm{F} = (F_1 \dots F_n)$, such that

$$c_{j,s}(\eta_j, s, \mathrm{F}) = \sum_{i=1}^{n} A_{j,s}(\eta_j, s) F_i$$

where $A_{j,s}(\eta_j, s) = a_i(\eta_j) s^q$, in which q is a positive exponent ensuring that larger individuals search a larger volume per unit time. The food intake is described using a type II functional response:

$$I_{j,s}\left(\eta_j, s, \mathrm{F}\right) = hs^m \frac{c_{j,s}\left(\eta_j, s, \mathrm{F}\right)}{hs^m + c_{j,s}\left(\eta_j, s, \mathrm{F}\right)}$$

Ingested food is assimilated with an efficiency ϵ_a. Assimilated energy is first used to cover metabolic maintenance costs:

$$E(\eta_j, s, \mathrm{F}) = \epsilon_a I_{j,s}(\eta_j, s, \mathrm{F}) - ks^p$$

where k is the size-specific constant of metabolic rate and p is the size-scaling exponent of the metabolic rate. Once metabolic maintenance costs are covered, a fraction ψ of the available energy is used for reproduction and the remaining for somatic growth:

$$G_j(\eta_j, s, \mathrm{F}) = \begin{cases} E(\eta_j, s, \mathrm{F})(1 - \psi(s, s_m)) & E(\eta_j, s, \mathrm{F}) > 0 \\ 0 & \text{otherwise} \end{cases}$$

A smooth step function is used to describe how energy allocated to growth gradually changes from 1 to 0 around the body size at maturation s_m (see Fig A):

$$\psi(s, s_m) = \left(1 + \left(\frac{s}{s_m}\right)^{-u}\right)^{-1} \left(\frac{s}{s_{\max}}\right)^{1-m}$$

In this equation, $s_{\max}$ is the maximum asymptotic body size, and u determines the width of the transition for energy allocation from growth to reproduction. According to this expression, when the maximum asymptotic body size is reached, an individual allocates all its available energy to reproduction. An individual therefore produces offspring at rate

$$B(\eta_j, s, \mathrm{F}) = \begin{cases} \frac{\epsilon_b}{s_b} E(\eta_j, s, \mathrm{F}) \left(\psi(s, s_m)\right) & E(\eta_j, s, \mathrm{F}) > 0 \\ 0 & \text{otherwise,} \end{cases}$$

where, ϵ_b is the efficiency at which assimilated energy is converted into offspring and s_b is the size at birth.

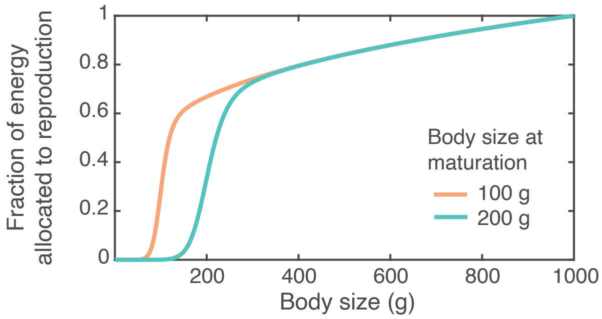

Figure A. Energy allocation and body size at maturation.
Energy allocation into reproduction (function ψ in Model 2) changes smoothly from 0 around the maturation size to 1 at the theoretical maximum asymptotic size (1000 g). Maturing at a small size enables an early onset of reproduction but reduces the growth rate. Because survival often increases with body size, the optimal body size at maturation depends on the tradeoff between reproductive investment and size-dependent survival. The code needed to generate this Figure can be found in https://zenodo.org/records/17049771.

Because larger organisms often have greater survival than smaller conspecifics (*42–53*) and this relationship scales exponentially with respect to body size (*54*), we assume mortality to decreases with body size following

$$\delta(s) = \delta_p e^{-s}.$$

Starvation mortality is not considered because at the ecological equilibrium, a population is viable (i.e. its density is positive) only if starvation conditions do not occur, i.e. if $E(\eta_j, s, \mathrm{F}) > 0$.

All assumptions discussed above pertain to the individual-level description. Without making further assumptions the population-level dynamics can be derived by bookkeeping following the Physiologically Structured Population Modeling approach[2]. The resulting ecological dynamics are:

$$\frac{dF_i}{dt} = \rho(F_{i\,\max} - F_i) - \sum_j \int_{s_\mathrm{b}}^{\infty} hs^m \frac{A_{j,s}(\eta_j, s)F_i}{\sum_k A_{j,k}(\eta_j, s)F_k + hs^m} N_j(t,s)ds,$$

$$\frac{\partial N_j(t,s)}{\partial t} + \frac{\partial G_j(\eta_j, s, \mathrm{F})N_j(t,s)}{\partial s} = -\delta(s)N_i(t,s),$$

and

$$G_j(\eta_j, s_b, \mathrm{F})N_i(t, s_b) = \int_{s_b}^{\infty} B(\eta_j, s, \mathrm{F})\, N_j(t,s)ds$$

as the boundary condition for the population reproduction rate. Parameter values for the life history description are determined from cross-species analysis of fish communities[1] and given in table B. Parameter values related to resource use are taken as in Model 1 (see Table A in S5 Text).

Table B. Life history parameters of Model 2

Parameter	Description	Value	Unit
	Diversifying lineage		
s_m	Body size at maturation	evolving trait	g
η	Feeding niche trait	evolving trait	-
D	Distance between optima to feed on the resources	1	-
τ	Width of the Gaussian curve describing the degree of specialization to successfully attack a food resource	1/3	-
α	Maximum size-specific attack rate	21.9	$m^3\ g^{-q}\ d^{-1}$
h	Maximum food intake	0.233	$g^{1-m}\ d^{-1}$
m	Exponent for maximum food intake	0.75	-
q	Size-scaling exponent for search volume	0.8	-
k	Standard metabolic rate	0.0274	$g^{1-p}\ d^{-1}$
p	Size-scaling exponent of metabolic rate	0.75	-
ϵ_a	Assimilation efficiency	0.6	-
u	Width of the transition for energy allocation from growth to reproduction	10	-
$s_{\max}$	Maximum asymptotic body size	1000	-
s_b	Body size at birth	1	g
ϵ_b	Efficiency of offspring production	0.1	-
δ_p	Size-dependent mortality rate	varied	d^{-1}
	Preexisting resources		
ρ	Renewal rate of preexisting resources	0.01	d^{-1}
ϕ	Total productivity of the habitat	varied	$g\ L^{-1}\ d^{-1}$
n	Number of basal resources in the habitat	2	-

Without making further assumptions the evolutionary dynamics can be obtained using standard methods of adaptive dynamics for size-structured populations[3]. These methods are incorporated into the PSPManalysis software package[4]. We use this package to determine whether diversification can occur or not for various levels of productivity and the body size at maturation, as well as the selection gradient of this life history trait (Fig 4B).

Model 3: Diversification and evolution of timing of diet shift

We adopt a minimal model to describe a size-structured population with a diet shift[5]. The model describes the dynamics of a population of individuals using different food resources in consecutive stages of their life history. For simplicity, we assume that the feeding rate on a food resource that is used only early in life is constant, such that there is no density dependence at this life stage. This may occur when the difference in the productivity between the resources used in different life stages is very large, causing density dependence to regulate the population dynamics only in the stage feeding upon the resource with the lowest productivity. In our case, this resource is the resource used late in life.

We consider an ecomorph population j that is composed of individuals characterized by their feeding niche trait η_j, body size s and body size at diet shift s_s. Individuals are born with size s_b and have access only to an early-life food resource, on which they feed at a rate f_b. They feed on this resource until they reach body size s_s, when they shift their diet and can exploit a variety of resources $\mathrm{F} = (F_1 \dots F_n)$. Juvenile individuals mature and start to reproduce at body size s_m. Individuals grow following

$$G(\eta_j, s, \mathrm{F}) = \begin{cases} \epsilon_g f_b & \text{if } s < s_s \\ \epsilon_g \displaystyle\sum_{i=1}^{n} a_i(\eta_j) F_i & \text{otherwise,} \end{cases}$$

and adults reproduce at a rate

$$B(\eta_j, s, \mathrm{F}) = \epsilon_b \sum_{i=1}^{n} a_i(\eta_j) F_i.$$

Mortality generally changes during life history. In particular, during an ontogenetic diet shift, organisms may experience such changes because a diet shift is often associated with a shift in habitat use and mortality may largely differ between habitats[6]. To reflect this, mortality is described as

$$\delta_s(s) = \begin{cases} \delta_1 & \text{if } s < s_s \\ \delta_2 e^{-s} & \text{otherwise.} \end{cases}$$

Using standard methods to formulate size-structured populations from individual life history processes[2] and after non-dimensionalising the model following ref[5], we obtain the set of equations that described the ecological dynamics:

$$\frac{dF_i}{dt} = (v - \phi F_i) - \sum_j \int_{w_s}^{\infty} \gamma_2(\eta_j, w, \mathrm{F})\, M_{2,j}(t, w) dw,$$

$$\frac{\partial M_{1,j}(t,w)}{\partial t} + \frac{\partial \gamma_1(f_b) M_{1,j}(t,w)}{\partial w} = -\varsigma_1 M_{1,j}(t,w)$$

$$\frac{\partial M_{2,j}(t,w)}{\partial t}+\frac{\partial \gamma_2\big(\eta_j,w,\mathrm{F}\big)M_{2,j}(t,w)}{\partial w}=-\varsigma_2(w)M_{2,j}(t,w),$$

and the boundary conditions for the diet shift

$$\gamma_2\left(\eta_j,w,\mathrm{F}\right)M_{2,j}(t,w_s)=\gamma_1(f_b)M_{1,j}(t,w_s)$$

and the population reproduction rate

$$\gamma_1(f_b)M_{1,j}(t,0)=\beta\,\gamma_2\left(\eta_j,w,\mathrm{F}\right)\int_1^{\infty}M_2(t,w)\,dw.$$

In this system, $M_{1,j}(t,w)$ and $M_{2,j}(t,w)$) are the density functions of the size distribution of the jth ecomorph population feeding on the early-life food resource and the food resources that provide ecological opportunity, respectively. This means that in the boundary between these density distributions, the diet shift occurs. The scaled body size variable w is related to the original body size measure following $w=(s-s_b)/(s_m-s_b)$, maturation hence occurs at $w=1, w_s$ is now the scaled-body size at diet shift. Additionally, the functions in the scaled model correspond to

$$\partial\gamma_1(f_b)=f_b,$$

$$\gamma_2\left(\eta_j,w,\mathrm{F}\right)=\sum_{i=1}^{n}a_i(\eta_j)F_i,$$

$$\varsigma_2(w)=\varsigma_c e^{-w}.$$

In the non-dimensionalised system, the parameters relate to the parameters of the individual life history description presented above according to Table C. We adopt the parameter values in ref[5] for the life history description (see Table C), and the parameter values related to resource use after the diet shift are taken as in Model 1 (see Table A in S5 Text).

Table C. Life history parameters of the non-dimensionalized Model 3

Description	Symbol	Relation with unscaled parameters	Value
Resource growth rate	ϕ	$\phi=\rho\sqrt{\frac{s_m-s_b}{\epsilon_g f_b}}$	0.01
Mortality before the diet shift	ς_1	$\varsigma_1=\delta_1\sqrt{\frac{s_m-s_b}{\epsilon_g f_b}}$	Varied (0.2, 0.25, 0.3)
Scaling factor of mortality after the diet shift	ς_c	$\varsigma_c=\delta_2\sqrt{\frac{s_m-s_b}{\epsilon_g f_b}}$	0.5

Adult fecundity scaled constant	β	$\beta = \frac{(s_m - s_b)\epsilon_b}{\epsilon_g}$	0.01
Body size at the diet shift	w_s	$w_s = \frac{s_s - s_b}{s_m - s_b}$	evolving trait

We use the same suit of approaches as in Model 2 to study the evolution of the feeding niche trait and the body size when individuals shift diet, which determines the timing of this shift. Therefore, the evolutionary dynamics can be obtained using standard methods of adaptive dynamics for size-structured populations[3]. We use the PSPManalysis software package[4] that implements these methods to determine whether diversification can occur or not for various levels of productivity and the body size at the diet shift, as well as the selection gradient of this life history trait (Fig 4C).

References

1. Hartvig, M., Andersen, K. H. & Beyer, J. E. Food web framework for size-structured populations. *J. Theor. Biol.* **272**, 113–122 (2011).

2. de Roos, A. M. A Gentle Introduction to Physiologically Structured Population Models. in *Structured-Population Models in Marine, Terrestrial, and Freshwater Systems* (1997). doi:10.1007/978-1-4615-5973-3_5.

3. Durinx, M., Metz, J. A. J. & Meszéna, G. Adaptive dynamics for physiologically structured population models. *J. Math. Biol.* **56**, 673–742 (2008).

4. de Roos, A. M. PSPManalysis: Steady-state and bifurcation analysis of physiologically structured population models. *Methods Ecol. Evol.* **12**, 383–390 (2021).

5. Chaparro-Pedraza, P. C. & de Roos, A. M. Ecological changes with minor effect initiate evolution to delayed regime shifts. *Nat. Ecol. Evol.* **4**, 412–418 (2020).

6. Sánchez-Hernández, J., Nunn, A. D., Adams, C. E. & Amundsen, P. A. Causes and consequences of ontogenetic dietary shifts: a global synthesis using fish models. *Biol. Rev.* **94**, 539–554 (2019).
