# Supplementary material for: Evolving life-history traits promote biodiversity via eco-evolutionary feedback mechanisms
Source: PLoS Biol. 2025 Nov 12;23(11):e3003492. doi: 10.1371/journal.pbio.3003492 (PMC12646416; doi:10.1371/journal.pbio.3003492)
Supplement: S3 Text — (PDF) [file pbio.3003492.s003.pdf]

Institute of Ecology and Evolution
University of Bern
Hochschulstrasse 6
3012 Bern
September 3rd, 2025

To the Editors of *PLOS Biology*

Dear Editors,

Thank you for your positive assessment of our manuscript. We greatly appreciate the comments and suggestions that we received, and we were happy to read that the reviewers appreciate our work. We have carefully revised the manuscript, trying to address each reviewer comment both in the response and via edits of the manuscript. You find the point-by-point responses below. We hope that you find the responses satisfactory and that the revised and amended manuscript will now be deemed suitable for publication in *PLOS Biology*.

Thank you for your consideration.

Sincerely,

Catalina Chaparro Pedraza

Claudia Bank

Reviewer #1:

The authors of "Life history evolution facilitates trophic diversification" demonstrate that life history evolution can strengthen intraspecific competition, which in turn promotes diversification. The authors set up a very important, interesting, and logical question in the introduction (with one caveat described in the next paragraph). The models appear well formulated and are described with admirable clarity, especially given their complexity. The text is extremely well written with appropriate information constrained to the supplement, but no issue following along with only the main text. The results also appear novel and are interesting. The discussion makes empirical connections quite well and raises important caveats. There is quite a bit to like about this study and, while I made many comments, the majority of them are in service of improving clarity and should be relatively straightforward to address.

My biggest concern is that the introduction makes it sound as if sympatric ecological trait diversification is a relatively easy process that is well established empirically. I think that this is somewhat oversold. Indeed, while theory (cited here) suggests that such adaptive speciation is plausible, other work suggests that the conditions under which branching an occur is more restricted (e.g., Gavrilets 2005 Evolution or Buerger & Schneider 2006 Am Nat). While this does not require a dramatic reframing, the authors should make clearer that there is some controversy around these claims (either in the introduction or discussion). This comment particularly applies to lines 24 and 30-31. It is worth noting that the authors use more balanced language at the beginning of the discussion.

We thank the reviewer for their positive feedback, their careful reading of the manuscript, and the helpful comments and suggestions. Following this first comment, we realized that our paragraph 2 in its previous version was not clear about the distinction between phenotypic diversification and speciation. We have thus rewritten this part to convey more clearly that phenotypic diversification may result in speciation only under limited conditions (lines 24-32).

Specific comments:

Line 7: The phrase "structural features" is not clear to me.

We now refer to “organismal properties” instead.

Line 41: I see what the authors are getting at here, but I think the statement is a bit ambiguous. The traits controlling resource acquisition in past models are not necessarily

subject to divergent selection. Indeed, under some conditions, selection remains stabilizing at equilibrium due to the assumption of a unimodal distribution of resources in the environment (e.g., Dieckmann & Doebeli 1999 Nature). This should be rephrased to be more accurate/nuanced.

We thank the reviewer for this accurate criticism of our terminology. We have rewritten this sentence accordingly, eliminating any reference to “subjected to divergent selection” (line 39-40).

Line 43: Typo, should say "strengthens".

We have corrected the typo.

Lines 78-79: A bit more explanation of why this is the appropriate measure of productivity in the model would be helpful.

The product of the carrying capacity and the supply rate of each resource correspond to its maximum possible production rate. Therefore, the sum of these products for all resources is an appropriate measure of the system productivity. We have rewritten the sentence to explain this in line 76-78.

Lines 86-87: It seems misleading to refer to this as a tradeoff because specialization on a single resource is only costly in the model (i.e., a narrower niche breadth does not confer a greater maximum attack rate but only reduces the attack rate on suboptimal resources). A similar comment applies on line 159.

We believe that the tradeoff as described by the reviewer may be different from the tradeoff in our model. The tradeoff in our model is defined by the way the attack rate on a food resource decreases as the feeding trait moves away from the optimal for this food resource. Because the optima are separated by a distance $D>0$, an increase in attack rate on one food resource necessarily entails a decrease in the attack rate on the other resources (explained in lines 85-86). This phenomenon has been referred to as a tradeoff by multiple theoretical and empirical studies (Ekkers et al., 2022; Hartvig et al., 2011; Herrel et al., 2009; Ware & Majerus, 2008), including studies on ecological diversification, e.g. (Patel & Schreiber, 2015; Rueffler et al., 2006). We thus follow the standard terminology and implementation in the field (lines 85-88).

Line 103: May be clearer as "that is, as the ratio . . ."

We have added the word “as”.

Lines 104-105: Would it be more appropriate to describe this as a birth rate?

We followed the suggestion of the reviewer and have replace fecundity by birth rate (lines 104-105).

Line 133: The term "simulated" is misleading in my opinion. This led me to believe the authors were considering individual-based simulations (as is often the case in models of adaptive speciation), rather than numerically integrating the differential equations in a deterministic model. Please rephrase to make this clearer.

We have replaced “simulated” by “computed” to avoid the reader to be misled (line 133).

Equation 3: It appears D is not defined in the main text

D is defined in line 80, where ecological opportunity and food resource is introduced in the model section.

Lines 176-177: It may be worth explicitly pointing out that similar intuition also applies for why there exists a minimum productivity for diversification to occur.

Following the reviewer’s suggestion, we have introduced a sentence in lines 187-188 to indicate that this intuition also applies for the productivity threshold: “Below this threshold of minimum productivity, the population density is low and intraspecific competition is weak, hindering diversification”.

Lines 180-181: This is a nice analytical result. It would be nice to add an explicit statement about what components of life history evolution affect condition 2 and in what direction. This can be pieced together by the reader from the information on line 167, but I think it would be more helpful to say it explicitly.

We appreciate that the reviewer shares our excitement about the results. We have extended our explanation in this paragraph to explicitly state what components (models parameters) affect condition 2 and the direction of the effect. Now in lines 188-192.

Figure 2 caption: "equilibriums" should instead be "equilibria".

We have corrected the typo.

Linen 205: I'm struggling to see how the sentence beginning with "As a consequence" follows from the previous sentence. Is it because, with so much resource depletion, selection favors trying to generalize and use both resources?

With strong resource depletion, the gain from becoming more specialized on the underused resource outweighs the loss from a decreased intake of the most used resource, driving the feeding niche trait towards an intermediate niche trait value between optima. We have now added this clarification to connect the previous sentence with the

following sentence stating that the niche trait corresponding to the intermediate between the optima is an attractor of the evolutionary dynamics (line 228-232).

Lines 247: It would be useful to cite figure 1 in this section since you give an overview of these models there.

We have introduced the reference to figure 1 in the first sentence of this section in the revised manuscript (line 274).

Lines 281-283: The focus on dispersal here and in figure 5 comes across as strange and tangential to the point of the study. I do not understand why dispersal is discussed alongside the modeling results given that it is not considered and that the results seem to stand on their own merits without this discussion of dispersal. I may be missing something, but I think some work is needed to better connect this text/figure to the modeling results.

We thank the reviewer for indicating their confusion with this part of the discussion. Our goal is to discuss our results in the broader context of biodiversity dynamics. Ecological diversification is common in complex landscapes composed of multiple ecosystems connected by dispersal (e.g., fish radiations across multiple lakes or bird radiations across island archipelagos). Although our model does not explicitly incorporate spatial structure, it assumes a colonization event at the very beginning, which is implicitly linked to dispersal across the landscape. In fact, ecological opportunity in many adaptive radiations has arisen through the colonization of newly formed ecosystems (e.g., islands or lakes) following dispersal events. Therefore, we consider that our results have implications for understanding biodiversity dynamics in complex landscapes, and that the discussion is the suitable place to point out such implications. We have added a clarification directly in the legend of figure 5 and rewritten the section in the discussion where we explain these broader implications (lines 312-315).

Lines 326-347: A very important caveat to point out that is well described by the authors.

Thank you for acknowledging our effort to describe the limitation of our modeling framework.

SI3 and SI4: "I" is used instead of "we" even though this is a multi-author paper.

We thank the reviewer for their careful reading of the manuscript and supplementary material. We have revised all text accordingly.

Suggestions that the authors should feel free to ignore:

Line 14: I find the parenthetical more distracting than helpful. The phrase "environmental conditions" is clear on its own.

We have changed the text accordingly.

Line 134: I found it confusing to have figures 2 and 3 cited here, before results are presented.

We have removed these references.

Lines 212-219: I'm not seeing that this paragraph really adds any new information that is not obvious from the previous paragraph. I think it could be cut, or if there are new pieces, merged with the previous paragraph or made more explicit.

The reviewer is correct that this paragraph contained some repetition. However, it also contained new information, in particular, it describes how the evolutionary attractor may be a local or global attractor depending on the life history trait value. This is important because when it is a global attractor, the initial condition (i.e. initial niche trait) does not affect the outcome, whereas if it is a local attractor diversification may or not may happen, depending on the initial condition. We have removed the repetitive part and merged the remaining information with the previous paragraph as suggested by the reviewer (lines 233-239).

Reviewer #2:

The manuscript "Life history evolution facilitates trophic diversification" describes how the adaptation of developmental traits, such as juvenile body size and the age of switch to an adult diet, affect the propensity for evolutionary branching in another trait that defines preferences for various resources. Three models that are considered point to the conclusion that the two-trait coevolution results in diversification of the trait related to resource preference. Some analytic results confirm and explain the outcomes of simulations.

We thank the reviewer for their detailed assessment of our work and the many helpful comments.

In my opinion, the work is interesting and most probably correct. However, the conclusions come as no surprise to anybody familiar with the body of literature on multitrait evolution, of which the Authors are apparently unaware. For example, in Ito, H. C., and U. Dieckmann. 2014. Evolutionary branching under slow directional evolution. Journal of Theoretical Biology 360:290-314. doi:10.1016/j .jtbi.2013.08.028. Ispolatov, I., V. Madhok, and M. Doebeli. 2016. Individual-based models for adaptive diversification in high-dimensional phenotype spaces. Journal of Theoretical Biology 390:97-105. It is studied under what conditions multidimensional phenotypes diversify "on the fly", not necessarily coming to a

convergent stable strategy with zero selection gradient. So the diversification of trait \eta in the direction orthogonal the ongoing adaptation of trait l (or other developmental traits in models 2 and 3) that happens for a certain interval of parameters does not come as something completely unexpected, but as yet another example of the fairly well-studied phenomenon.

We thank the reviewer for bringing up these references, which are indeed very relevant to our work, and for indicating that we need to more clearly compare our work with the classical adaptive-dynamics literature. We have now included these references in our discussion. However, we disagree with the reviewer's assessment of the novelty of our results: none of their mentioned studies describe the phenomenon that we are describing in the present manuscript. The mentioned studies all use the same assumption regarding resource distribution as classic adaptive dynamics models. In our revised manuscript version, we have emphasized that, in contrast to our model, classic adaptive dynamics models assume a single resource, which causes the condition for convergence stability to be always satisfied (line 339-340). Indeed, the two papers referred by the reviewer acknowledge that, in their model, evolution always drives the ancestral population to the unique optimal phenotype. In contrast to these studies, we describe here the effect of an additional phenotypic trait (i.e. the life history trait) on the outcome of diversification in a model with multiple resources and thus with multiple phenotypic optima for the ancestral population. We show that adaptive evolution along the axis of this additional phenotypic trait can facilitate diversification in the trait defining food preference (i.e. trait eta), and that this is mediated by the effect of the additional trait on the condition for convergence stability. This is because, different from the classical models assuming a single resource, the condition for convergence stability is not always satisfied in our model (see condition 2 in section "By altering the stability of evolutionary equilibria, life history influences the conditions for diversification"). Therefore, the mechanism driving our findings is fundamentally different from the mechanisms investigated in the papers cited by the reviewer.

Moreover, the results are also different. For example, Ispolatov, Madhok, and Doebeli (2016) and Doebeli and Ispolatov (2014) showed that adaptive diversification becomes less likely when the phenotype space is high-dimensional than when it has one dimension because the likelihood that an evolutionary trajectory converges to an evolutionary branching point decreases with increasing dimensionality. Conversely, our results show that evolution of a secondary trait enables the feeding niche trait to converge to the evolutionary branching point. In fact, figure 2 shows that, when the phenotype space is unidimensional (figure 2A and 2B), diversification does not occur, whereas, when the phenotype space is two-dimensional (figure 2C and 2D), diversification does occur. Our

results, which show that the addition of a secondary phenotypic trait facilitates diversification, thus contrast previous studies indicating that a higher dimensional phenotype space hinders diversification. The reason for this difference is the mechanism underlying our findings, which, as we explained above, stems from our model assumption of multiple resources that fundamentally departs from the single resource assumption in classical adaptive dynamics models. To avoid confusion about this, we now emphasize more clearly in the discussion that diversification models considering multiple traits as well as more classical single trait models cannot generate the observed phenomena described in our work (see line 344-346).

So I suggest to tune down the emphasis on the novelty of interplay between the evolution of those two traits, but to rather focus on better linking the model to empirical data (e.g. making the distribution of food resources more realistic, explaining and justifying the functional forms and parameter intervals , showing the actual trajectories of trait evolution, etc).

We thank the reviewer for the suggestion. Our models indeed cover diversification in a more realistic distribution of food resources than previous diversification models. Specifically, previous diversification models considering multiple traits, e.g., the models cited by the reviewer, assume a single resource, although natural adaptive radiations were shown to typically involve diversification to exploit a variety of different dietary resources. Therefore, our assumption of multiple resources is both novel in the body of research studying diversification in multi-trait space and it is also more aligned with empirical patterns. We have clarified this alongside our edits following the previous comment (see line 346-351).

Another general recommendation is to unify the terminology applied to evolution of l and \eta. After all, both are evolving under "natural selection" and in principle can undergo branching. The use of terms specific for the evolution of l and \eta creates unnecessary confusion as if two different processes are at play.

We thank the reviewer for this valid comment. We have revised the manuscript to refer to evolution of both traits as driven by natural selection throughout.

Below are several more specific suggestions:

Lines 29-31, In a realistic high-dimensional phenotype space, branching can occur on a fly without convergence to a fitness minimum, refs above

These lines have been removed from the revised manuscript based on the suggestions of reviewer 1.

Line 32 Predation?

We were not 100% sure what the reviewer was trying to point out here. We hypothesized that they refer to predation as another factor that could drive ecological diversification, which is not addressed in our manuscript. To address this potential confusion, we have edited this sentence (line 33).

What is the difference between the "ecological diversification process" and "natural selection" (line 39)? Isn't the former just a specific case of the latter? I think the whole paragraph (35-45) is totally misleading.

Natural selection is the driver of ecological diversification. However, natural selection can also drive other processes that do not result in ecological diversification, for example, adaptation without diversification. We thus ask whether natural selection acting on a life history trait subjected to stabilizing selection can facilitate diversification via natural selection on the niche trait. Following the suggestion of reviewer 1, who also pointed out their confusion with some of the wording in this paragraph, we have rewritten a large fraction of this paragraph (lines 37-43).

Lines 97-98 The following phrase in not clear, "Hence, somatic growth only increases when the assimilated food exceeds the metabolic cost "

The function $\phi(\eta,F)$ that defines the somatic growth is a maximum between 0 and the difference between the assimilated food and the metabolic cost. If this difference is negative, then somatic growth takes the value of 0. This prevents that an individual shrinks in size when the metabolic cost is higher than the assimilated food. We have added this clarification in line 97.

In the definition of Model 1 it has to be clearly stated that the juveniles don't actually grow, but rather stay as juveniles with the constant size l until maturing into adults at the rate specified by (101)

We use a size-based, stage-specific model, which allows to represent the dynamics of a population in terms of a low-dimensional system of ordinary differential equations, but nonetheless faithfully represents the dynamics of a population model in terms of partial differential equations involving a full size-distribution (De Roos, et al. Simplifying a physiologically structured population model to a stage-structured biomass model. Theor. Popul. Biol. 73, 47–62 (2008)). Therefore, although our model is based on the dynamics of densities of individuals within each life stage, the maturation function depends on the growth rate of a juvenile individual as well as on the range of sizes over which it must grow. Therefore, in our stage-structured model, the dynamics of maturation are dictated by the individual growth process of juveniles. We have added a sentence clarifying this in line 104.

Lines 148-149 "For diversification to occur, theory proposes that a population must experience disruptive selection for a significant amount of time (13)" What is changing with time?

Rueffler et al (2006), in their seminal paper "Disruptive selection and then what?", identified two types of disruptive selection: "In the first type, which does not lead to diversification, selection prevents a population from experiencing such a situation for any significant amount of time". This is the case when a trait value at which selection is disruptive is not an attractor of the evolutionary dynamics, or in other words when the condition for convergence stability is not satisfied. In the second type of disruptive selection, diversification can occur because selection drives the population towards the trait value where selection is disruptive. Our analysis builds upon this seminal work and adopts their definition of disruptive selection leading to diversification, referencing them accordingly (reference 12 in line 151).

Statements like "when the directional selection ceases" (line 137) are ambiguous as it is not clear to what directional selection, in l or \eta, they apply.

Following the reviewer's comment, we have clarified our wording throughout the manuscript. The new version in this instance reads: "when directional selection on the feeding niche trait ceases" (lines 138-139).

Lines 161-163 Here again, this has to be re-worded as the evolving phenotype is 2-dimensional and convergence to an attractor is not a necessary condition for diversification.

Previous works, for example, Ispolatov, Madhok, and Doebeli (2016) and Doebeli and Ispolatov (2014), have indeed shown that convergence to an attractor is not a necessary condition for diversification under the assumption of such models. A core assumption in these previous models is that both traits control resource acquisition. Different from these models, in our models only one of the traits controls resource acquisition, that is the feeding niche trait, eta. The life history trait, in turn, affects the demography of the population but not resource acquisition. Therefore, although our model considers two evolving phenotypic traits, only one alters resource acquisition, which is the source of disruptive selection. Therefore, the conditions for diversification for systems with a unidimensional phenotype space also hold in our case. We have added a clarification explaining this in lines 153-156.

Line 188 What if the first scenario were started with l close to its diversification-promoting value (1.5, line 196?)?

The trait l does not have a diversification-promoting value because it is under stabilizing selection. It is possible that reviewer refers to the niche trait eta instead. In that case, the scenario mentioned by the reviewer is the scenario that Rueffler et al (2006) defined as the first type of disruptive selection, in which the condition for convergence stability is not satisfied. In this scenario, the population evolves away from the value of disruptive selection, and only if its starting value is exactly equal to the value at which selection is disruptive will it experience disruptive selection, which is a degenerate case of little biological relevance.

Lines 220-235 This it a totally predictable outcome. In my opinion, its description should not constitute a dedicated subsection.

We respectfully disagree with this assessment by the reviewer. Although our previous results indeed suggest that that the speed at which life history traits evolve affects the timing of diversification, the magnitude of this effect on the ensuing diversity is not straightforward to predict. Therefore, have decided to keep this subsection in the main text.

Lines 249-250 "In model 2, we study the effect of the evolution of body size at maturation, which determines the transition of energy allocation from growth to reproduction (figure S1). If it refers to ":w", why not to write it? I

Model 2 is different from Model 1, in which w refers to the size at maturation. In Model 2, we consider the full size distribution, and not only the stage structure, of a population. Model 2 is based on the food web bioenergetic approach for size-structured populations introduced by Hartvig et al (2011). In the model description (in SI6), we define all model parameters and evolving traits of Model 2, including body size at maturation, denoted by the symbol s_m. Because the model details for Model 2, including the notation of the evolving traits, are presented in the supplementary material, we prefer not to use the symbol in the main text to avoid confusing readers.

Line 255. The definition of Model 3 should be way more quantitative.

We appreciate the reviewer's concern about the compact description of Model 3. However, our goal was to make the main text comprehensible for the broad readership of Plos Biology despite the different models' intricacies and the mathematical complexity. Model 3 is therefore quantitatively defined in the supplementary material SI6, such that the detailed description is available for any quantitatively inclined reader.

Both models 2 and 3 should be properly defined and analyzed in the main text, or completely placed in the supplementary materials.

As explained in our response to the previous comment, our goal was to stay concise and clear. Moreover, the analysis of both Model 2 and Model 3 does not result in novel findings beyond the results we obtained from Model 1. This is an important part of our study: these results confirm the results from Model 1, but given that the model structure and assumptions are very different, they allow us to generalize our results beyond the specific assumptions of Model 1. Including the detailed description of Models 2 and 3 in the main text would increase the length of the manuscript by about seven pages. Therefore, we decided to briefly describe Model 2 and Model 3 in the main text and to leave the model details to the supplementary material.

Does the size of juveniles actually change with time in models 2 and 3? If so, there should be equations that describe it, or a statement saying otherwise.

We thank the reviewer for this comment, which pointed out to us that a key feature of Models 2 and 3 was not clear from the main text of the previous version of our manuscript. In Model 2 and in Model 3, the individual growth rate is explicit because we follow the dynamics of the population's full size distribution, and not only the density of life stages as in the Model 1. As a result, the population dynamics are represented by a system of partial differential equations, which we present in SI6. Besides presenting the population-level equations in SI6, we provide an overview of the individual-level processes, including juvenile growth in body size, with their corresponding equations. To avoid confusion in the main text, we have added a clarification to indicate that individuals actually grow in Model 2 (line 277) and in Model 3 (line 284). Additionally, the legend of figure 1 now states that body size changes over an individual's lifetime in Model 2 and 3.

Lines 338-341. I cannot follow this argument. How the competition among juveniles is different from that among adults? With properly set parameters, the mechanism for diversification should work universally.

We thank the reviewer for pointing out their confusion with this section. The competition between juveniles and adults can be very different if individuals at different stages use different food resources. This is a very well-studied phenomenon (see e.g. Schreiber & Rudolf, 2008; ten Brink & de Roos, 2017), with counterintuitive consequences for ecological diversification, including the effect recalled in line 376 that was first shown by (Ten Brink & Seehausen, 2022). In this section of the discussion, we acknowledge that such counterintuitive consequences do not emerge in our model because it does not consider the possibility that competition differs among individuals at different life stages. This is a limitation that we acknowledge in the discussion because it implies that our model does not cover the eco-evolutionary dynamics of species with complex life cycles, which are common across animal taxa (lines 361-382).

There should be more explanations to the figures. What do colours and black arrows mean in Fig. 1G?

We have made changes to figure 1 and its caption to clarify the meaning of the arrows. We have also revised the other figures accordingly.

in the simulation described in Fig. 3, are both strains present at the beginning of simulation?

We seeded 2 lineages, each with one ancestral population: one with initial niche trait 0.8 and the other one with initial niche trait 10.2. We have explained this in the legend of the figure to clarify.

It is hard to reconcile the fairly broad yellow areas in Fig. 4 with the statement that diversification is only happening when the selection gradient in \eta becomes zero. It would help to see the actual trajectories of various strains, possibly for both l and \eta coordinates.

We thank the reviewer for this comment. Figure 4 shows in the top panels the selection gradient of the offspring size (in A), the selection gradient of the maturation size (in B), and the selection gradient of the body size at diet shift (in C). This figure shows that, in the neighborhood of the optima of these life history traits (vertical purple line), diversification can occur for a larger range of productivities than if the life history trait is far from its optimum. Figure 4 does not show the selection gradient for the niche trait eta, and therefore it does not make any reference to a value of zero on the selection gradient of the niche trait. To link the evolutionary trajectories in figure 2 with the corresponding fitness landscape, we now have added a line to figure 4A indicating the transect that corresponds to the fitness landscape in figure 2. This way, we explicitly link figure 4 and actual evolutionary trajectories as suggested by the reviewer.

In the Discussion, it is worth noting once again that the observed phenomena are a particular case of diversification in a two-dimensional trait space.

The observed phenomena, namely the fact that life history evolution can facilitate diversification, is the consequence of how life history traits influence the condition for convergence stability, which is not always satisfied in our model. Because this condition is always satisfied in existing diversification models with a two- or higher-dimensional trait space, our study presents and discusses a different class of models that cannot be considered a particular case of the existing body of multi-trait adaptive-dynamics models. To avoid confusion about this, we now emphasize more clearly in the discussion that diversification models considering multiple traits as well as more classical single trait

models cannot generate the observed phenomena described in our work (see line 340-346).

In Fig. 5, how the dispersal phase is related to diversity or diversification?

Figure 5 is part of the discussion, intending to present how the process described in this study is integrated into more complex biodiversity dynamics where diversification is one of the processes occurring in a complex landscape with multiple habitats (e.g. multiple lakes for fish or multiple islands for birds). In these dynamics, the colonization of new habitats, where diversification takes place, is fueled by dispersal. Multiple recent studies have shown that dispersal in turn is affected by diversity via altered ecological interactions that exert selection on dispersal-related traits (see ref 75-80 in the main text). We now clarify the connection between dispersal and diversity in line 312-313 and extend the explanation in the figure legend, contextualizing our results more broadly in the topic of biodiversity dynamics as explained above.

Reviewer #3:

The manuscript "Life history evolution facilitates trophic diversification" presents a compelling theoretical exploration of how life history evolution contributes to the emergence of ecological diversity. Using a suite of adaptive dynamics models, the authors demonstrate that natural selection acting on life history traits (such as offspring size, age at maturation, and timing of diet shift) enhances intraspecific competition, thereby creating conditions that facilitate niche diversification. Their models consistently show that life history adaptation reduces the environmental thresholds (e.g., productivity) needed for diversification and accelerates its occurrence. By integrating life history theory with ecological diversification models, the study provides a novel framework to explain how organismal traits shape biodiversity in a self-reinforcing feedback loop. This work is timely, well-structured, and theoretically robust, with potential to spark empirical research testing the macroevolutionary implications of life history evolution.

We thank the reviewer for sharing their excitement with our study, and for their detailed and explicit comments.

The following are areas for improvement:

1. Overextension of claims regarding empirical generality: While the theoretical results are strong, several claims about empirical implications (e.g., links between offspring size evolution and species richness) seem to be overstated. For example, references to

salmonids and ray-finned fishes (lines 299-300) should be tempered, as these remain hypotheses without direct causal tests linking life history evolution to diversification.

We agree with the reviewer that the findings correspond to hypotheses that remain to be tested. Therefore, we have rewritten our statements in order to temper the tone. For example, the sentence introducing the salmonids example now reads: “The case of Salmonids may qualitatively support this prediction” in line 329. Similarly, the sentence closing the paragraph has been changed and now reads: “Our results may thus provide an explanation linking fast life history evolution, rapid local adaptation, and propensity to diversify”.

2. Insufficient biological intuition: Some model descriptions, particularly in the results for Model 1 (lines 90-132), are highly technical and would benefit from additional biological interpretation. For example, the ecological meaning of compound parameters like E and G (line 166) could be clarified for a general audience.

We thank the reviewer for pointing out that intuition was lacking in the description of some of the model parameters. We have included biological explanations for the parameters that did not have such an explanation in our previous version, such as the parameter gamma. Additionally, we have now explained the biological meaning of the compound variables E and G in lines 180-184.

3. Overreliance on SI: While mathematical derivations are expected in a theoretical biology paper, the heavy reliance on SI for core insights (e.g., conditions for diversification) may reduce accessibility. Including intuitive summaries of key equations (e.g., Eq. 3-4) and trade-off implications in the main text would strengthen the presentation.

We acknowledge that much of our work is presented in the SI for conciseness of the main text. To address the reviewer’s comment, we have extended the description of eq. 3, providing further explanation of the implications of the tradeoff that underpins condition 1 (lines 166-170). We have also extended the explanation of eq. 4 by including a more detailed explanation of the simple parameters (line 188-191) and the compound variables E and G (see above), and we have restructured the paragraphs that include the explanations regarding the implications of eq. 4 to improve readability. We hope that these changes improve accessibility of the main results while maintaining conciseness and readability of the manuscript.

Minor suggestions:

Line 16: "Our results call for a better integration..." - consider specifying which research fields should be integrated (e.g., "integrating empirical life history studies with theoretical models of niche diversification").

We have rewritten this sentence to more clearly state that we believe the fields of life history evolution and niche diversification need more integration (line 16-17).

Line 46: Add a reference or brief explanation to support the claim that life history evolution alters intraspecific competition. Also, here the authors refer to "insights" (plural) but provide a singular reference.

We included reference 21 to support this claim. The words “key insights” have been changed to “a key insight” (line 44).

Line 94-95: Clarify the biological significance of parameter γ scaling juvenile foraging relative to adults.

We thank the reviewer for pointing out that this need clarification. To better explain the biological relevance of the parameter gamma, we have added the following sentence in line 93-95: “The general rule across animal taxa is that larger organisms have higher foraging capacity than smaller conspecifics, and include references. To meet this rule in the model, the parameter gamma<1 scales the juvenile feeding rate, such that it is lower than that of the adults”.

Line 107-110: The assumption that juvenile mortality decreases exponentially with offspring size should be justified with a brief biological rationale and a couple of references to the relevant literature.

We now explain that the relationship between mortality and body size scales exponentially with respect to body size, providing a biologically grounded support to our assumption (line 110). We also included a reference to support this statement (ref 55 Glazier 2025).

Line 146: "...which we used to calculate the fitness landscape" - clarify how this is done in a more intuitive way, e.g., linking population structure to fitness gradients.

We have now specified that we use the quantities listed in line 146-147 to calculate the fitness gradient and the curvature of the fitness landscape (using eq. SI1.4-SI1.6).

Line 178-179: "Life-history evolution... affects Condition 2" - better explain why only this condition (and not mutual invasibility) is altered by life history traits.

Following the reviewer’s comment, we have included a sentence linking the now expanded explanations of Condition 1 to this section (line 198-200). This sentence now clarifies why only Condition 2 is affected by life history evolution.

Line 221-223: Spell out how mutation rate differences were implemented—does this mimic variation in evolvability or genetic variance?

We now specified that the differences in mutation rate represent differences in evolvability (line 247-248).

Line 259-260: Include more biological background on the trait "timing of diet shift" - e.g., which taxa exhibit it and how it links to ecological diversification.

We have added biological background regarding the life history trait studied in Model 3 as suggested by the reviewer (line 281-283).

Line 267: "The three models greatly differ..." - consider summarising in a short table or schematic the main features of Models 1-3 for clarity.

Figure 1 present a scheme contrasting the main differences between the models. It emphasizes the different life history traits that evolve in each model and schematically shows its link with the model structure. It also emphasizes the differences in terms of the population ecological dynamics, as in Model 1 we deal with a population structured by life stage and in Model 2 and 3 with a population structured by body size. Therefore, in our opinion, figure 1 conveys the main features in which the models differ. We have added a reference to figure 1 at the end of this sentence (line 298).

Line 296-297: "Our results provide an explanation linking fast life history evolution..." - a citation here is required to help ground this speculative bridge between theory and observation.

We have moderated the strength of this statement by introducing "may". The new version reads "Our results may provide an explanation linking fast life history evolution, rapid local adaptation, and propensity to diversify", thus indicating that our statement is a hypothesis.

Line 310-313: "...preclude the possibility..." - this sentence could be clearer by explicitly stating that single-resource models inherently satisfy convergence stability and therefore mask life history effects.

We thank the reviewer for the suggestion. It improves the clarity of this sentence and also addresses some of Reviewer 2's comments (line 344-346).

Line 348-349: Consider explicitly stating that this study is among the first to formally integrate life history theory with diversification models.

We have modified the sentence according to the suggestion (line 383).
